# Hepatitis E Virus in Yellow Cattle, Shandong, Eastern China

**DOI:** 10.3201/eid2212.160641

**Published:** 2016-12

**Authors:** Bingyu Yan, Li Zhang, Lianfeng Gong, Jingjing Lv, Yi Feng, Jiaye Liu, Lizhi Song, Qing Xu, Mei Jiang, Aiqiang Xu

**Affiliations:** Shandong University, Jinan, China (B. Yan, L. Zhang, J. Lv, Y. Feng, J. Liu, L. Song, Q. Xu, A. Xu);; Shandong Center for Disease Control and Prevention, Jinan (B. Yan, L. Zhang, J. Lv, Y. Feng, J. Liu, L. Song, Q. Xu, A. Xu);; Yantai Center for Disease Control and Prevention, Yantai, China (L. Gong, M. Jiang)

**Keywords:** hepatitis E, hepatitis E virus, viruses, zoonoses, yellow cattle, cow, livestock, phylogeny, transmission, reservoir, China

**To the Editor:** Hepatitis E, caused by hepatitis E virus (HEV), is recognized as a zoonosis (*1*)*.* HEV has been identified in a wide range of animals, and swine is the primary reservoir (*2*). In cattle, HEV strains have been recently described in yak (*3*), Holstein cows and their milk (*4*), and dairy cows in Xinjiang Province, China (*5*), but not in other cattle. Yellow cattle (*Bos taurus*), the predominant breed (≈80%) in China (*6*), widely distributed over the country, and commonly used for meat and milk production or as a draft animal, could act as a potential HEV reservoir. The objective of this study was to determine whether HEV strains are circulating among yellow cattle in Shandong Province of eastern China.

During April–November 2011, a total of 842 blood samples from yellow cattle of local breeds were collected monthly as part of a severe fever with thrombocytopenia syndrome virus study. These samples were obtained from Laizhou and Penglai Counties (≈100 km apart) of Yantai Prefecture in Shandong Province.

Because the prevalent seasons for human HEV in this region were winter and spring, 254 samples (Laizhou = 131; Penglai = 123) collected only in April and November were selected for detection of HEV. All 254 cattle appeared to be healthy. Sixteen were <1 year of age, 108 were 1–3 years of age, and 130 were >3 years of age. The cattle came from 20 villages (10 villages per county) and were raised by the local peasants, who owned an average of 2 cattle (range 1–8). The animals were bred mainly to produce meat and seldom to produce milk. 

Additional serum samples from domestic sheep, dogs, and chickens were also collected in this region simultaneously (Technical Appendix Table 1). All blood samples were centrifuged, and the separated serum was stored at −70°C until use. The protocol for animal sampling was approved by the Animal Care Committee of the Chinese Center for Disease Control and Prevention.

We tested serum samples for total antibodies against HEV by using a double-antigen sandwich ELISA kit (Wantai Biological, Beijing, China) that uses a recombinant peptide of HEV open reading frame 2 (aa 394–606) from the virus as the antigen (*7*). Overall, the proportion seropositive for antibodies against HEV in yellow cattle was 47% (120/254; 95% CI 41%–54%), in line with the 28.2% positivity ratio previously reported in cattle from 26 provinces of China (*8*), suggesting that a high proportion of yellow cattle were exposed to HEV in this region. The proportions seropositive among sheep, dogs, and chickens were 32% (70/222), 41% (80/194), and 8% (41/484), respectively (Technical Appendix Table 1).

We used nested reverse transcription PCR to amplify 644 nt within HEV open reading frame 2 region, as described previously (*9*). We detected HEV RNA in 8 of 254 cattle samples; the overall proportion seropositive was 3%. Positive yellow cattle included one <1 year of age, three 1–3 years of age, and four >3 years of age. The 8 sequences obtained in this study (GenBank accession nos. KU904271, KU904273, KU904274, KU904278–KU904282) were subjected to phylogenetic analysis along with reference sequences for subtyping (*10*).

Using MEGA 7.0 software (http://www.megasoftware.net) with the maximum-likelihood algorithm and a bootstrap of 1,000 replicates, we constructed a phylogenetic tree (Technical Appendix Figure). All 8 sequences clustered within subtype 4d of HEV. The sequences were similar to each other (95.5%–99.8% similarity in nucleotide sequence) and similar to sequences reported for other cattle (83.3%–85.3%; Technical Appendix Figure). Moreover, these sequences shared 96.1%–96.6% similarity with a human HEV strain (GenBank accession no. KC163335) from the Yantai Prefecture in 2012 and 95.7%–97.9% similarity with a swine strain (GenBank accession no. KF176351) isolated in Shandong Province the same year.

Our data strongly indicate that HEV infection occurs in yellow cattle and that they could also play a role as a reservoir of HEV. Because these animals serve mainly as a source of food, consumption of undercooked meat from yellow cattle, similar to pork, might also contribute to the transmission of HEV to humans. Additionally, we also detected HEV RNA in 8 of 70 sheep (Technical Appendix Table 2). Eight sequences from yellow cattle had 95.1%–99.8% nt homology with 8 sheep-derived HEV strains, possibly because mixed raising of domestic livestock is popular in this region. Our finding of high sequence similarity between yellow cattle, sheep, swine, and human populations suggests a complicated interspecies transmission of HEV occurred in this province. Further studies are required to evaluate the contribution of the yellow cattle reservoir to human HEV infection.

Technical Appendix Hepatitis E virus testing in yellow cattle, sheep, dogs, and chickens, Shandong Province, China, 2011.
