# Supplementary material for: Hepatitis E Virus in Yellow Cattle, Shandong, Eastern China
Source: Emerg Infect Dis. 2016 Dec;22(12):2211–2. doi: 10.3201/eid2212.160641 (PMC5189148; doi:10.3201/eid2212.160641)
Supplement: Technical Appendix — Hepatitis E virus testing in yellow cattle, sheep, dogs, and chickens, Shandong Province, China, 2011. [file 16-0641-Techapp-s1.pdf]

Article DOI: http://dx.doi.org/10.3201/eid2212.160641

Hepatitis E Virus in Yellow Cattle, Shandong, Eastern China

Technical Appendix

Technical Appendix Table 1. Proportion of domestic yellow cattle, sheep, dogs, and chickens seropositive for anti-hepatitis E virus, Shandong Province, China, 2011*

Sample category	Age, y	No. samples	No. positive	Proportion seropositive for anti-HEV %, (95% CI)
Yellow cattle	<1	16	5	31 (11–59)
	1–3	108	43	40 (31–50)
	>3	130	72	55 (46–64)
	Total	254	120	47 (41–54)
Sheep	<1	83	25	30 (21–41)
	1–3	103	32	31 (22–41)
	>3	36	13	36 (21–54)
	Total	222	70	32 (25–38)
Dogs	<1	32	15	47 (29–65)
	1–3	113	49	43 (34–53)
	>3	49	16	33 (20–48)
	Total	194	80	41 (34–49)
Chickens	–	484	41	8 (6–11)

* Blood samples of each species were obtained monthly during April–November 2011, as part of a severe fever with thrombocytopenia syndrome virus study. The total number of samples collected in April and November from domestic yellow cattle, sheep, dogs, and chickens were 254, 222, 194, and 484, respectively. Anti-HEV total antibodies were detected by a double-antigen sandwich ELISA kit (Wantai Biological, Beijing, China). Signal-to-cutoff values ≥1 were considered positive. HEV, hepatitis E virus.

Technical Appendix Table 2. Hepatitis E virus–positive strains isolated from domestic yellow cattle, sheep, dogs, and chickens, Shandong Province, China, 2011*

Source	Location	Age, y	GenBank accession no.
Yellow cattle	Penglai	>3	KU904273
	Penglai	1–3	KU904274
	Penglai	1–3	KU904282
	Laizhou	>3	KU904271
	Laizhou	<1	KU904278
	Laizhou	1–3	KU904279
	Laizhou	>3	KU904280
	Laizhou	>3	KU904281
Sheep	Penglai	>3	KU904272
	Laizhou	1–3	KU904267
	Laizhou	1–3	KU904268
	Laizhou	1–3	KU904269
	Laizhou	1–3	KU904270
	Laizhou	<1	KU904275
	Laizhou	<1	KU904276
	Laizhou	1–3	KU904277
Dogs†	–	–	–
Chickens†	–	–	–

* All 254 cattle and HEV total antibody–positive serum samples from sheep (n = 70), dogs (n = 80), and chickens (n = 41) were analyzed for HEV RNA. Positive results were obtained by reverse transcription PCR. HEV, hepatitis E virus.
† No samples from dogs or chicken were positive for HEV RNA.

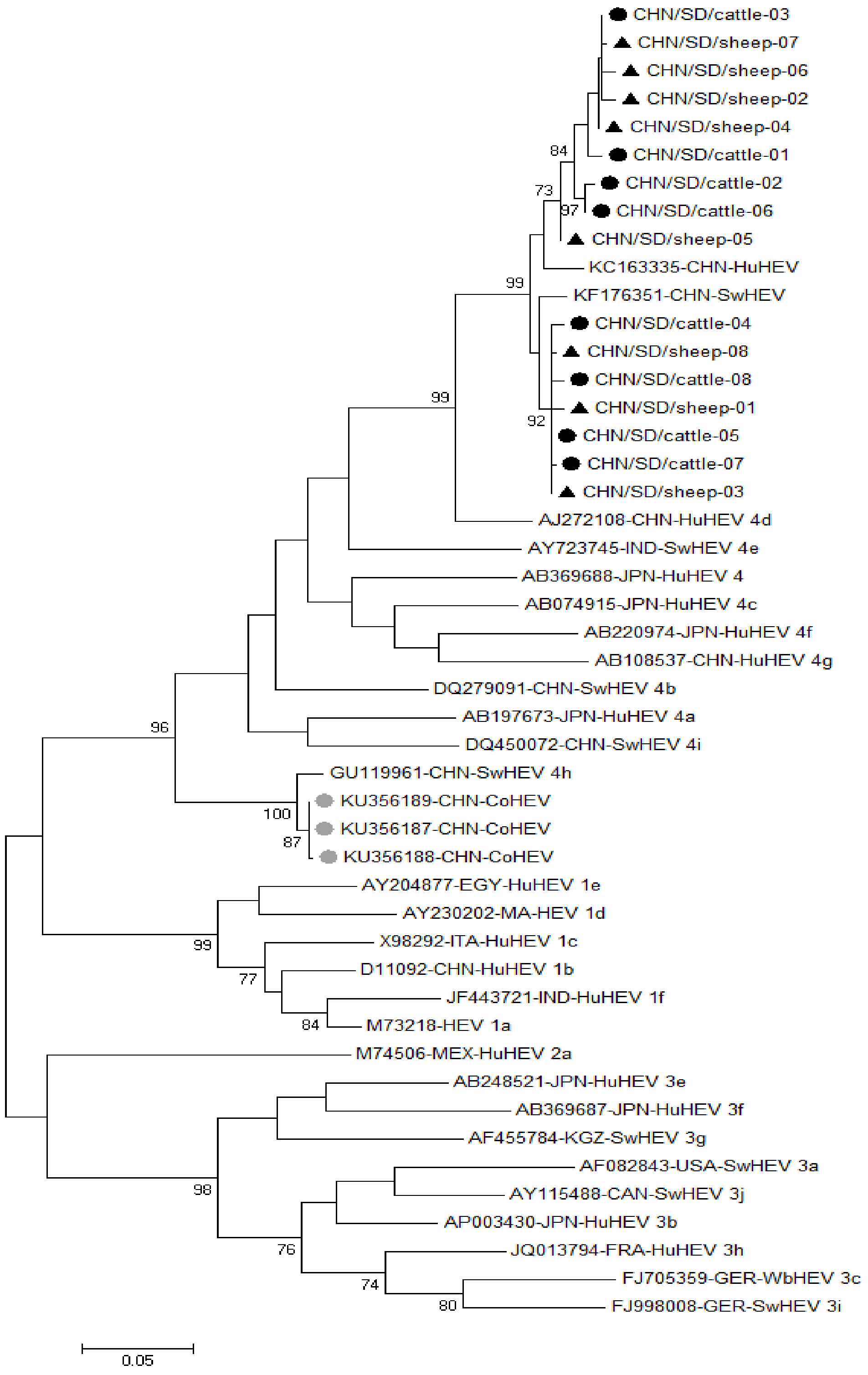

Technical Appendix Figure. Phylogenetic analysis of hepatitis E virus strains based on the 540-nt open reading frame 2 fragment (positions 5765–6304 of reference sequence M73218). The phylogenetic tree was constructed with MEGA 7.0 software (http://www.megasoftware.net) by using the maximum-likelihood algorithm and Jukes-Cantor model with 1,000 bootstrap replicates. Bootstrap values (%) >70

are indicated at branch nodes. HEV sequences derived from cattle and sheep in this work are indicated with black circles and triangles, respectively. The reference sequences included in the figure were the ones suggested by Smith et al. 2016 for genotype subtyping. We also included for comparison HEV sequences isolated from cattle in other studies (gray circles). CAN, Canada; CHN, China; Co, cow; EGY, Egypt; FRA, France; GER, Germany; Hu, human; IND, India; ITA, Italy; JPN, Japan; KGZ, Kyrgyzstan; MA, Morocco; MEX, Mexico; Sw, swine; USA, United States of America; Wb, wild boar. All sequences obtained in this study are deposited in GenBank (accession nos. KU904267–KU904282). Scale bar represents nucleotide substitutions per site.
